# Thermotolerance improvement of engineered *Saccharomyces cerevisiae ERG5 Delta ERG4 Delta ERG3 Delta*, molecular mechanism, and its application in corn ethanol production

**DOI:** 10.1186/s13068-023-02312-4

**Published:** 2023-04-12

**Authors:** Peizhou Yang, Wenjing Wu, Jianchao Chen, Suwei Jiang, Zhi Zheng, Yanhong Deng, Jiuling Lu, Hu Wang, Yong Zhou, Yuyou Geng, Kanglin Wang

**Affiliations:** 1 School of Food and Biological Engineering, Anhui Key Laboratory of Intensive Processing of Agricultural Products, Hefei University of Technology, 420 Feicui Road, Shushan District, Hefei, 230601 Anhui China; 2 Department of Biological, Food and Environment Engineering, Hefei University, 158 Jinxiu Avenue, Hefei, 230601 China; 3 Suzhou Cofco Biochemical Co., Ltd., Suzhou, 234001 China; 4 Hefei Knature Bio-Pharm Co., Ltd., Hefei, 231131 China

**Keywords:** Thermotolerant improvement, *Saccharomyces cerevisiae*, CRISPR–Cas9 approach, Transcriptomics, Ethanol production, Ergosterol, Corn

## Abstract

**Background:**

The thermotolerant yeast is beneficial in terms of efficiency improvement of processes and reduction of costs, while *Saccharomyces cerevisiae* does not efficiently grow and ferment at high-temperature conditions. The sterol composition alteration from ergosterol to fecosterol in the cell membrane of *S. cerevisiae* affects the thermotolerant capability.

**Results:**

In this study, *S. cerevisiae ERG5*, *ERG4*, and *ERG3* were knocked out using the CRISPR–Cas9 approach to impact the gene expression involved in ergosterol synthesis. The highest thermotolerant strain was *S. cerevisiae ERG5ΔERG4ΔERG3Δ*, which produced 22.1 g/L ethanol at 37 °C using the initial glucose concentration of 50 g/L with an increase by 9.4% compared with the wild type (20.2 g/L). The ethanol concentration of 9.4 g/L was produced at 42 ℃, which was 2.85-fold of the wild-type strain (3.3 g/L). The molecular mechanism of engineered *S. cerevisiae* at the RNA level was analyzed using the transcriptomics method. The simultaneous deletion of *S. cerevisiae ERG5*, *ERG4*, and *ERG3* caused 278 up-regulated genes and 1892 down-regulated genes in comparison with the wild-type strain. KEGG pathway analysis indicated that the up-regulated genes relevant to ergosterol metabolism were *ERG1*, *ERG11*, and *ERG5*, while the down-regulated genes were *ERG9* and *ERG26*. *S. cerevisiae ERG5ΔERG4ΔERG3Δ* produced 41.6 g/L of ethanol at 37 °C with 107.7 g/L of corn liquefied glucose as carbon source.

**Conclusion:**

Simultaneous deletion of *ERG5*, *ERG4*, and *ERG3* resulted in the thermotolerance improvement of *S. cerevisiae ERG5ΔERG4ΔERG3Δ* with cell viability improvement by 1.19-fold at 42 °C via modification of steroid metabolic pathway. *S. cerevisiae ERG5ΔERG4ΔERG3Δ* could effectively produce ethanol at 37 °C using corn liquefied glucose as carbon source. Therefore, *S. cerevisiae ERG5ΔERG4ΔERG3Δ* had potential in ethanol production at a large scale under supra-optimal temperature.

**Supplementary Information:**

The online version contains supplementary material available at 10.1186/s13068-023-02312-4.

## Background

Fungus *Saccharomyces cerevisiae* (*S. cerevisiae*) is of great importance in various biotechnological applications with unique biological characteristics of fermentation capacity, ethanol production, and CO_2_ release [1]. In the bioethanol industry, any small efficiency improvement of ethanol production using thermotolerant yeasts would be economically significant [2, 3]. Bioethanol is generally produced by *S. cerevisiae* at comparatively low temperatures (30–32 °C) owing to its limited thermotolerance [4]. In addition to *Saccharomyces cerevisiae* growing at a normal temperature, there are also yeasts in nature that can tolerate higher temperatures, such as *Ethanol Red* [5, 6]. The proteome and transcriptome approaches revealed that the strain could consume glucose for ethanol production at 35 °C under the conditions of Erg13 and Gsy1 overexpression with the cumulative increase of trehalose [5, 6]. The construction and domestication of thermotolerant yeast have important application value.

The comparatively high temperature meets the requirement in the release of fermentable sugars from lignocellulosic biomass [3] like starch-based feedstocks [7], lignocellulosic *Conocarpus erectus *[8], and rice husk [9]. Additional cooling devices and power were used to meet the temperature requirement of the growth and fermentation of *S. cerevisiae*. In addition, high temperature enables more efficient feedstock hydrolysis, thus resulting in ethanol productivity increase in simultaneous saccharification and fermentation [10, 11]. Thus, thermotolerant yeasts contribute to reducing cooling and distillation costs and decreasing contamination chances during the fermentation process of ethanol production [12, 13].

**Fig. 1:**
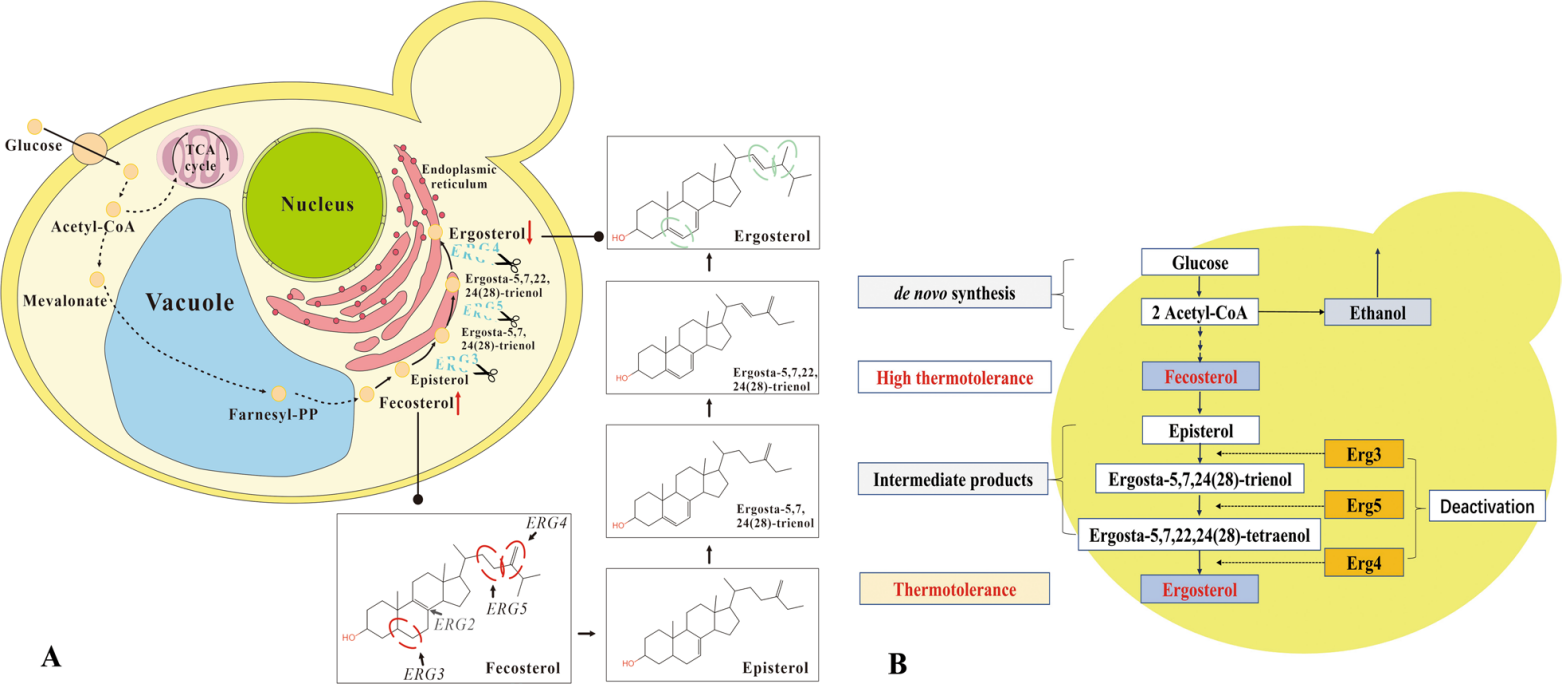
De novo ergosterol biosynthesis and strategy for ergosterol composition alteration. **A** De novo ergosterol biosynthesis via pathways of glucose, acetyl-CoA, fecosterol, and ergosterol; **B** ergosterol composition alteration by deactivation of Erg3, 4, and 5 enzymes during fecosterol and ergosterol in this study

Thermotolerant capability of yeast could be improved by various factors of sterols [14], heat shock proteins [15], trehalose [16], and glycerol [17] in cell membranes. Sterols are the essential structural and regulatory components of cell membranes in *S. cerevisiae *[18]. The predominant sterol, ergosterol, is the main precursor of cortisone and the hormone progesterone in yeasts [19]. De novo ergosterol biosynthesis is a highly complex energy-consuming pathway involving the participation of more than 20 *ERG* enzymes [20] (Fig. 1). Sterol composition alteration from ergosterol to fecosterol renders yeast thermotolerance using genome-wide gene expression and metabolic-flux analysis approaches [21]. In the highly thermotolerant yeast mutant, the accumulation concentration of fecosterol was far higher than that in the wild-type yeast [21], which meant that fecosterol could play a more important role in yeast thermotolerance than ergosterol. Fecosterol in *S. cerevisiae* was formed from zymosterol catalyzed by C-24 sterol methyltransferase (Erg6p) [22]. Fecosterol is then converted into ergosterol in the endoplasmic reticulum via the catalysis of C-8 sterol isomerase (Erg2), C-5 sterol desaturase (*ERG3*), C-24 (28) sterol reduce (*ERG4*), and C-22 sterol desaturase (*ERG5*) [20].

The deletion of single *ERG3, ERG4*, and *ERG5* genes in *S. cerevisiae* contributed to the thermotolerance improvement of yeast [23, 24]. However, the thermotolerant capability of different sterol compositions from ergosterol to fecosterol is still not well known. In this study, the thermotolerance performances of different mutation combinations of *ERG3*, *ERG4*, and *ERG5* in *S. cerevisiae* were compared using the Clustered Regularly Interspaced Short Palindromic Repeats–Cas9 (CRISPR–Cas9) approach (Fig. 1). The molecular mechanism of engineered *S. cerevisiae* strain was explored using the transcriptomics approach. Further, the conversion of corn hydrolysate into ethanol by engineered *S. cerevisiae* was investigated to estimate the large-scale application feasibility under high-temperature conditions.

## Results

### Construction of engineered* S. cerevisiae* strains

**Fig. 2:**
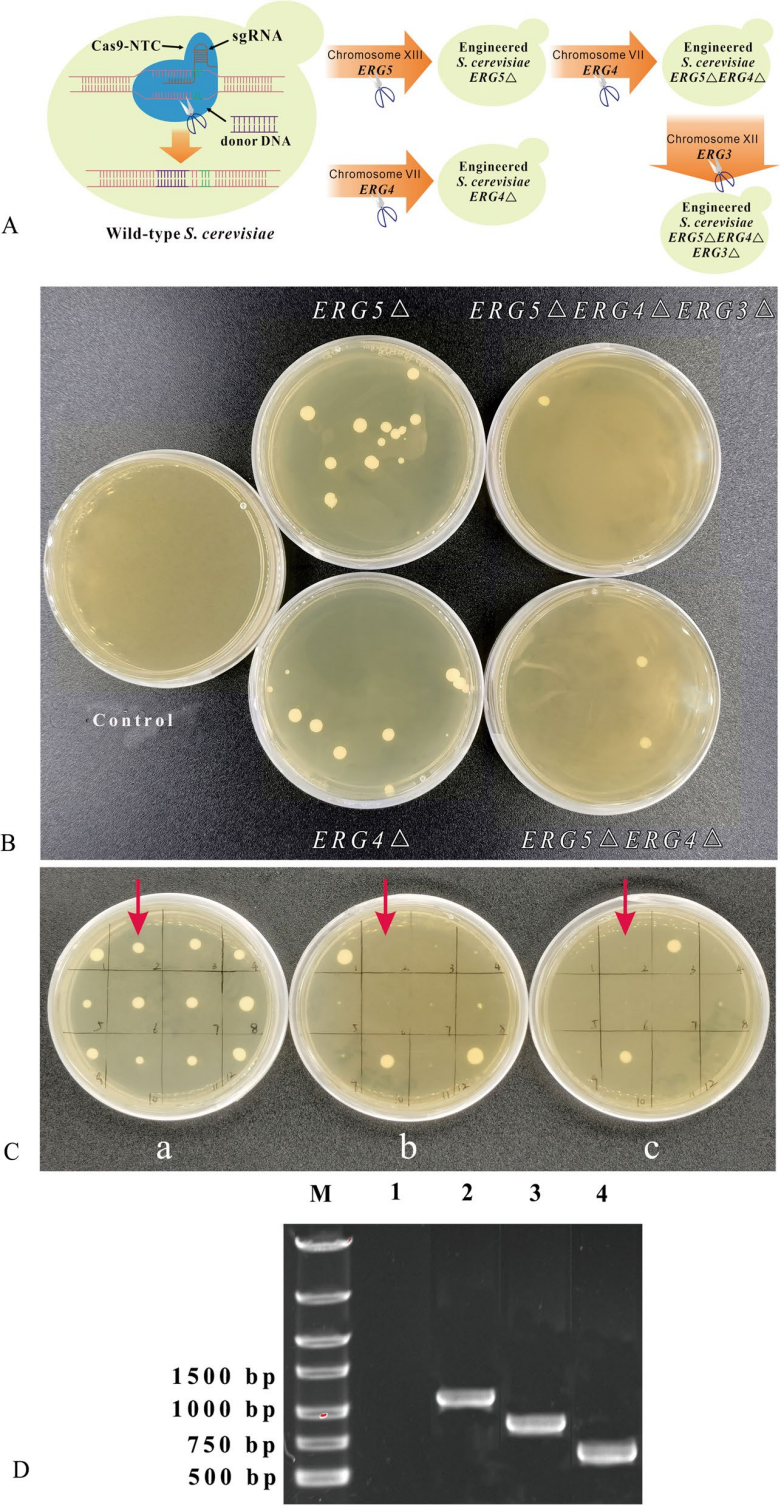
Screening of the putative transformants and molecular identification of *S. cerevisiae ERG5ΔERG4ΔERG3Δ.* **A** Construction strategy of four engineered *S. cerevisiae* strains; **B** Screening of four putative transformants on solid plates containing two antibiotics, while the wild-type strain could not grow on the screening plate; **C** Screening of vector loss of *S. cerevisiae ERG5Δ* transformants for further transformation of *ERG4*. The cell proliferation of *S. cerevisiae ERG5Δ* transformant after transformation of ERG5-gRNA-trp-HyB and Cas9-NTC was carried out via the liquid fermentation approach. The proliferative colonies were screened and cultured on YPD plate a for 48 h at 30 °C. Then, the colonies on plate a were transferred to the corresponding positions on plates b and c. The media in a, b, and c were YPD medium, YPD medium containing 80 μg/mL of nourseothricin (NTC), and YPD medium containing 300 μg/mL of hygromycin B (HyB), respectively. The colonies (arrow) could not grow on both b and c, which meant that the corresponding colony on plate a had lost both ERG5-gRNA-trp-HyB and Cas9-NTC; **D** *S. cerevisiae ERG5ΔERG4ΔERG3Δ* identification by amplification of *XYNA* (lane 2, 1129 bp), *CEL* (lane 3, 880 bp), and *MFC* (lane 4, 628 bp). Lane M and 1 represented DNA Marker and the control, respectively

In this study, *S. cerevisiae ERG5*, *ERG4*, and *ERG3* were, respectively, deleted via the insertion of *MFC*, *XYNA*, and *CEL* using the CRISPR–Cas9 knocking-out approach (Fig. 2). Four yeast mutants were named by *S. cerevisiae ERG5Δ*, *ERG4Δ*, *ERG5ΔERG4Δ*, and *ERG5ΔERG4ΔERG3Δ* based on the specific knock-out genes (Fig. 2A). *S. cerevisiae ERG5Δ*, *ERG4Δ*, *ERG5ΔERG4Δ*, and *ERG5ΔERG4ΔERG3Δ* transformants were screened on the double-antibiotic media, while no colony cultured on the media for the wild-type strain (Fig. 2B). The putative transformants were further identified on the YPD solid medium according to the double-antibiotic method of hygromycin B and nourseothricin after simultaneous transformation of Cas9-NTC and gRNA vectors (Fig. 2C). The putative *S. cerevisiae ERG5ΔERG4ΔERG3Δ* was further identified by the amplification of insertion DNA (Fig. 2D). After gene sequencing, the putative transformants were finally confirmed to be true transformants.

### Effect of gene deletion on the growth of engineered *S. cerevisiae* strains

**Fig. 3:**
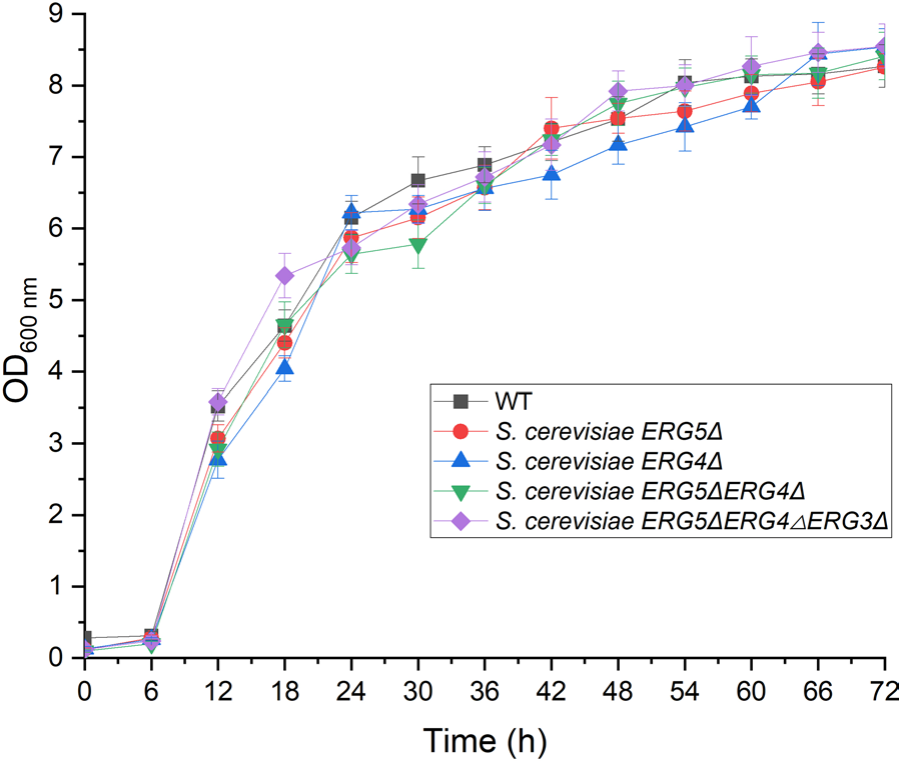
OD_600 nm_ values of four engineered *S. cerevisiae* strains during fermentation

The contents of yeast cells were determined by the absorbance values at the wavelength of 600 nm (OD_600 nm_). OD_600 nm_ values of *S. cerevisiae ERG5Δ*(8.26), *ERG4Δ*(8.54), *ERG5ΔERG4Δ*(8.41), and *ERG5ΔERG4ΔERG3Δ*(8.55) were 1-, 1.03-, 1.02-, and 1.03-fold in comparison with the wild-type strain (8.27) (Fig. 3). Thus, all the engineered *S. cerevisiae* mutants after gene deletion still kept good growth and proliferation capability.

### Thermotolerance of engineered *S. cerevisiae* strains on the solid YPD medium

**Fig. 4:**
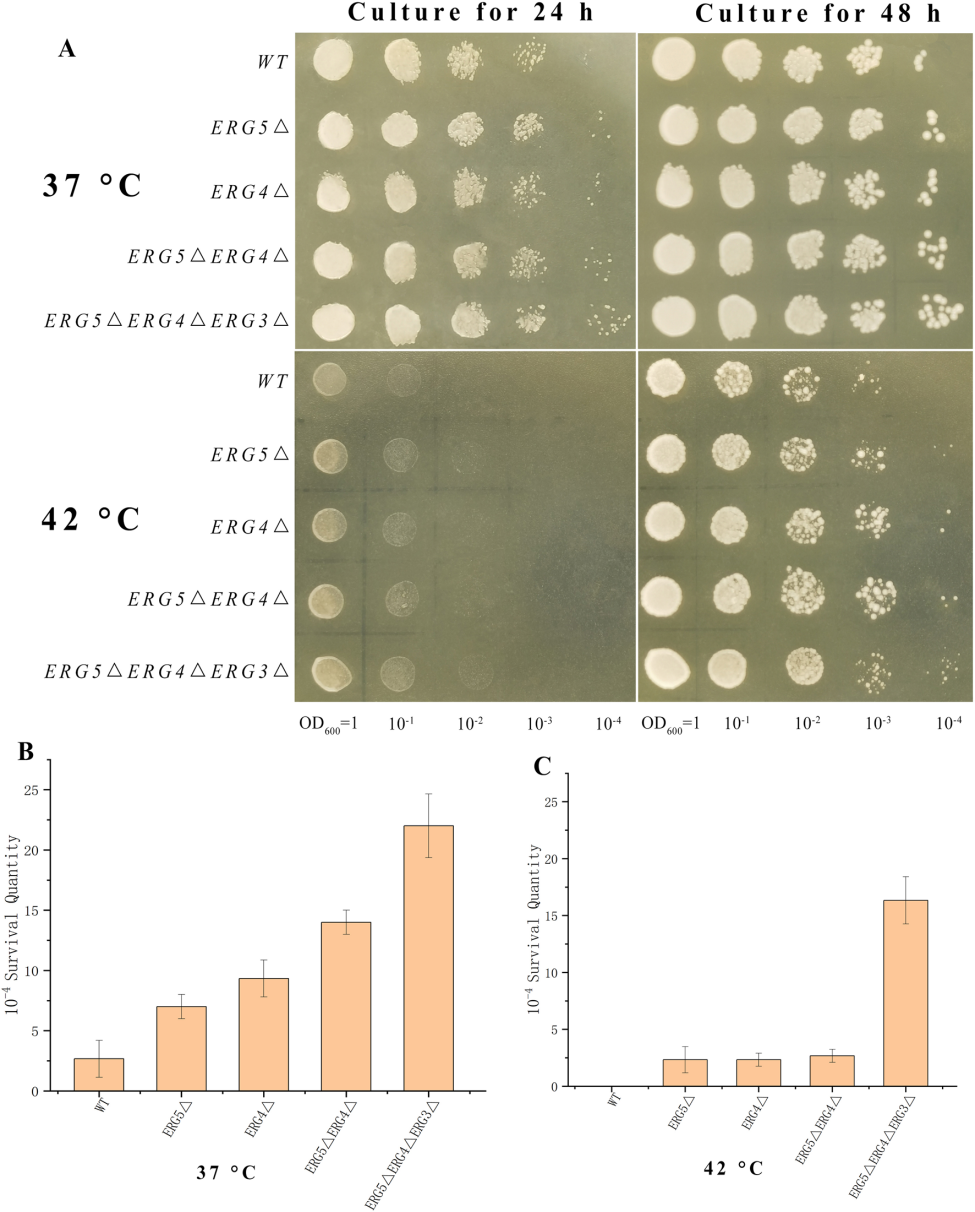
Thermotolerance investigation of engineered *S. cerevisiae* strains at 37 and 42 °C. **A** Temperature tolerance of engineered strains at different concentrations and temperatures; **B**, **C** Colony numbers of each strain after dilution of 10^–4^ OD_600 nm_ under the conditions of 37 and 42 °C, respectively

The thermotolerance of four engineered *S. cerevisiae* strains on solid YPD medium was investigated under the conditions of 37 and 42 °C (Fig. 4). The colony plaque area and density of strain at 37 °C were higher than those at 42 °C. After a dilution of 10^–4^, the colonies of the wild-type and engineering strains were detected on the solid YPD medium. The colony numbers from *S. cerevisiae ERG5Δ*(5), *ERG4Δ*(8), *ERG5ΔERG4Δ*(14), and *ERG5ΔERG4ΔERG3Δ*(23) were 1.7-, 2.7-, 4.7-, 7.7-fold in comparison with the wild-type strain (3), respectively. In addition, after culture for 24 h at 42 °C, the growth of all the strains was severely inhibited. However, after culture for 48 h, most of the strains grew on the solid medium. The density and quantity of colonies from engineered yeasts were higher than the wild-type strain. Under a dilution of 10^–4^, many colonies from engineered yeasts were detected on the plate; meanwhile, no colony from the wild-type strain was detected. Thus, *S. cerevisiae ERG5ΔERG4ΔERG3Δ* represented the strongest thermotolerance capacity among engineered *S. cerevisiae* strains.

### Cell viability of *S. cerevisiae ERG5ΔERG4ΔERG3Δ* under high temperature

**Fig. 5:**
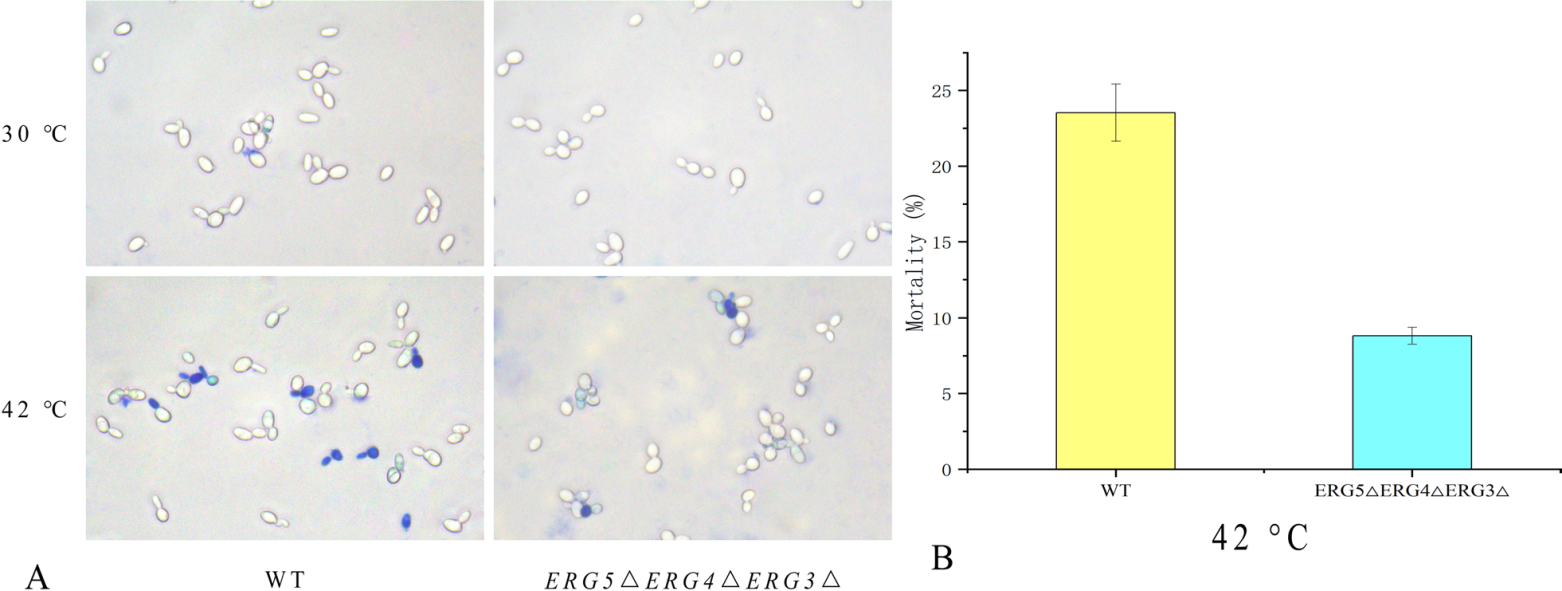
Investigation of the cell activity of *S. cerevisiae ERG5ΔERG4ΔERG3Δ* at 30 and 42 °C. **A** Cell morphology of *S. cerevisiae ERG5ΔERG4ΔERG3Δ* and wild-type strain after dyeing at 30 and 42 °C; **B** mortality percentages of *S. cerevisiae ERG5ΔERG4ΔERG3Δ* and wild-type strain at 42 °C

The cell viability was investigated by the morphology observation of *S. cerevisiae ERG5ΔERG4ΔERG3Δ* at 30 and 42 °C based on the tinting color degree after dyeing of methylene-blue solution (Fig. 5). The living and dead yeast cells were differentiated based on the tinting color after the dyeing. The dye cannot be adsorbed on the cell wall of living yeast, while the surface of dead cells of yeast is easy to adhere dye. After a treatment of 30 °C for 3 h, both *S. cerevisiae ERG5ΔERG4ΔERG3Δ* and wild-type strain maintained good cell viability. After treatment at 42 °C for 3 h, the mortality percentage of *S. cerevisiae ERG5ΔERG4ΔERG3Δ* (8.8%) was considerably lower than the wild-type strain (23.5%). Therefore, *S. cerevisiae ERG5ΔERG4ΔERG3Δ* with the simultaneous deletion of *ERG5*, *ERG4*, and *ERG3* possessed improved thermotolerance.

### Glucose consumption and ethanol production at 30, 37, and 42 °C

**Fig. 6:**
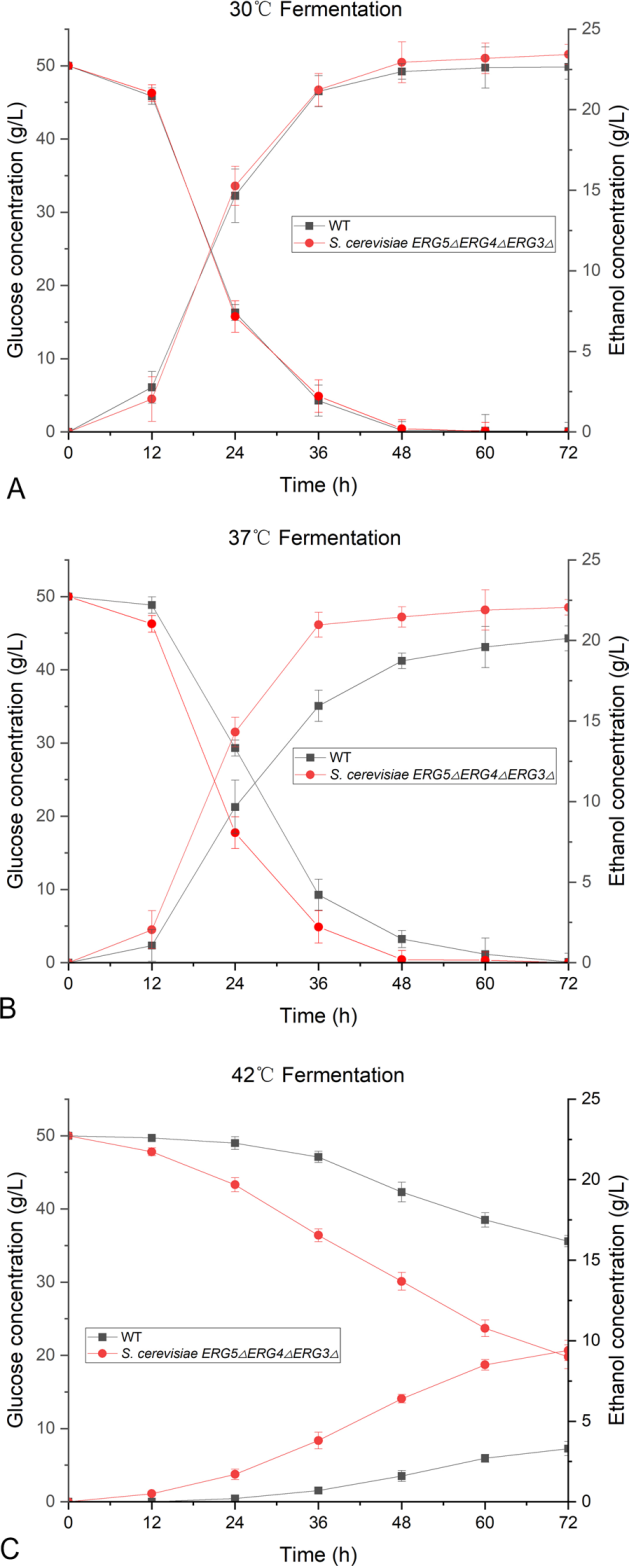
Glucose consumption and ethanol production of *S. cerevisiae ERG5ΔERG4ΔERG3Δ* under temperatures of 30 °C (**A**), 37 °C (**B**), and 42 °C (**C**)

The glucose consumption and ethanol production of *S. cerevisiae ERG5ΔERG4ΔERG3Δ* were investigated using the wild-type strain as the control at 30, 37, and 42 °C (Fig. 6). At 30 °C, *S. cerevisiae ERG5ΔERG4ΔERG3Δ* and wild-type strain had almost the same trends of glucose consumption and ethanol production with 50 g/L glucose as the initial concentration (Fig. 6A). The ethanol concentration from *S. cerevisiae ERG5ΔERG4ΔERG3Δ* was 23.4 g/L after fermentation of 72 h, which was slightly higher than that of the wild-type strain (22.7 g/L). The deletion of *ERG5*, *ERG4*, and *ERG3* did not affect glucose consumption and ethanol production under regular fermentation conditions.

The ethanol production of *S. cerevisiae ERG5ΔERG4ΔERG3Δ* at 37 ℃ was higher (22.1 g/L) than that of the wild-type strain (20.2 g/L) (Fig. 6B). At 42 °C, both cell proliferation and ethanol production of engineered strain were severely inhibited during fermentation. After fermentation for 72 h, the ethanol conversion rate of glucose was 0.31 g/g at 42 °C, which was 1.35-fold in comparison with the wild-type strain (0.23 g ethanol/g glucose) (Fig. 6C). Therefore, *S. cerevisiae ERG5ΔERG4ΔERG3Δ* still maintained a high yield of ethanol at the super optimal temperature compared with the wild-type strain.

### Expression density distribution and differential expression genes (DEG)

Gene expression density distribution and DEG were analyzed based on the data from transcriptomics sequencing of *S. cerevisiae ERG5ΔERG4ΔERG3Δ* (Additional file 1). After cDNA library construction and sequencing, gene expression density distribution of *S. cerevisiae ERG5ΔERG4ΔERG3Δ* was compared with the wild-type strain (Additional file 1A). Both *S. cerevisiae ERG5ΔERG4ΔERG3Δ* and wild-type strain possessed the same density from purple area. In addition, the densities from red and blue areas were from *S. cerevisiae ERG5ΔERG4ΔERG3Δ* and wild-type strain, respectively. TPM density distribution from *S. cerevisiae ERG5ΔERG4ΔERG3Δ* was lower than the wild-type strain.

Scatter plot was used to reflect the difference of 2170 genes in total between the wild-type strain and *S. cerevisiae ERG5ΔERG4ΔERG3Δ* (Additional file 1B). *S. cerevisiae ERG5ΔERG4ΔERG3Δ* represented 278 up-regulated genes and 1892 down-regulated genes in comparison with the wild-type strain. The number of down-regulated genes (6.8-fold) was far higher than up-regulated genes, which indicated that the simultaneous deletion of three genes of *ERG5*, *ERG4*, and *ERG3* could cause the reduction of overall cell metabolism in engineered yeast.

### Gene ontology (GO) annotation of DEG

**Fig. 7:**
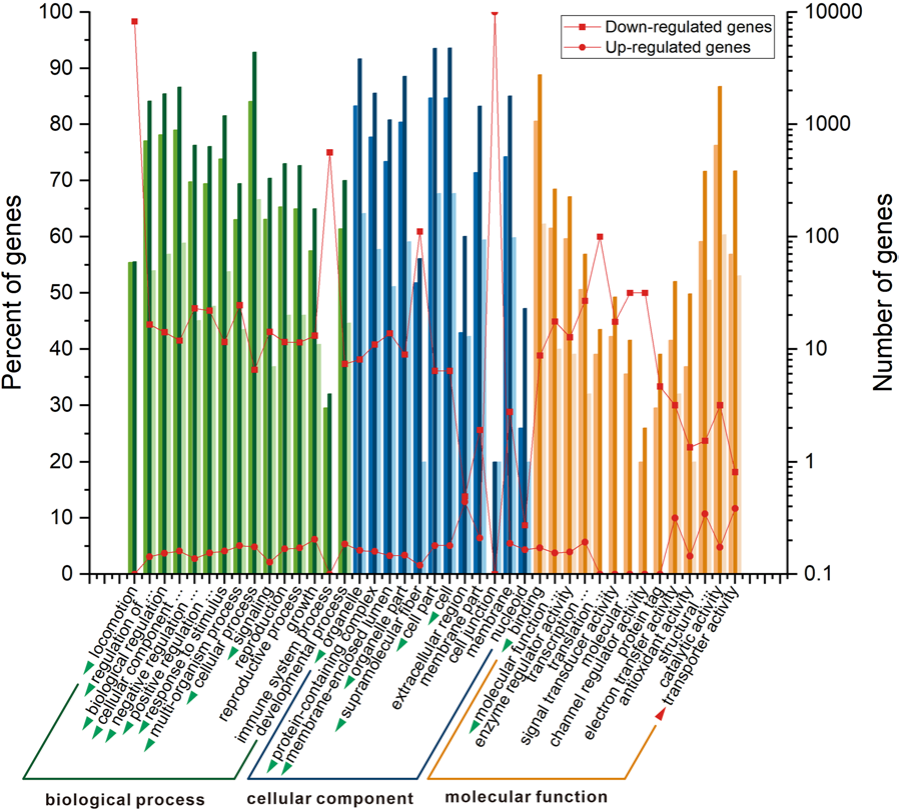
GO annotation of DEG between *S. cerevisiae ERG5ΔERG4ΔERG3Δ* and the wild-type strain

In this study, GO was classified into three categories of biological process (15 items), cellular component (12 items), and molecular function (14 items) between *S. cerevisiae ERG5ΔERG4ΔERG3Δ* and the wild-type strain. The categories of significant differences in gene expression were investigated using the number and percent of up-regulated and down-regulated genes in each item (*p* < 0.05) (Fig. 7). The numbers of tested genes in each category also had significant differences (*p* < 0.05). Most of the gene categories exhibited down-regulated characteristics. Overall, *S. cerevisiae ERG5ΔERG4ΔERG3Δ* after gene deletion resulted in down-regulated expression of genes.

### KEGG enrichment analysis

**Fig. 8:**
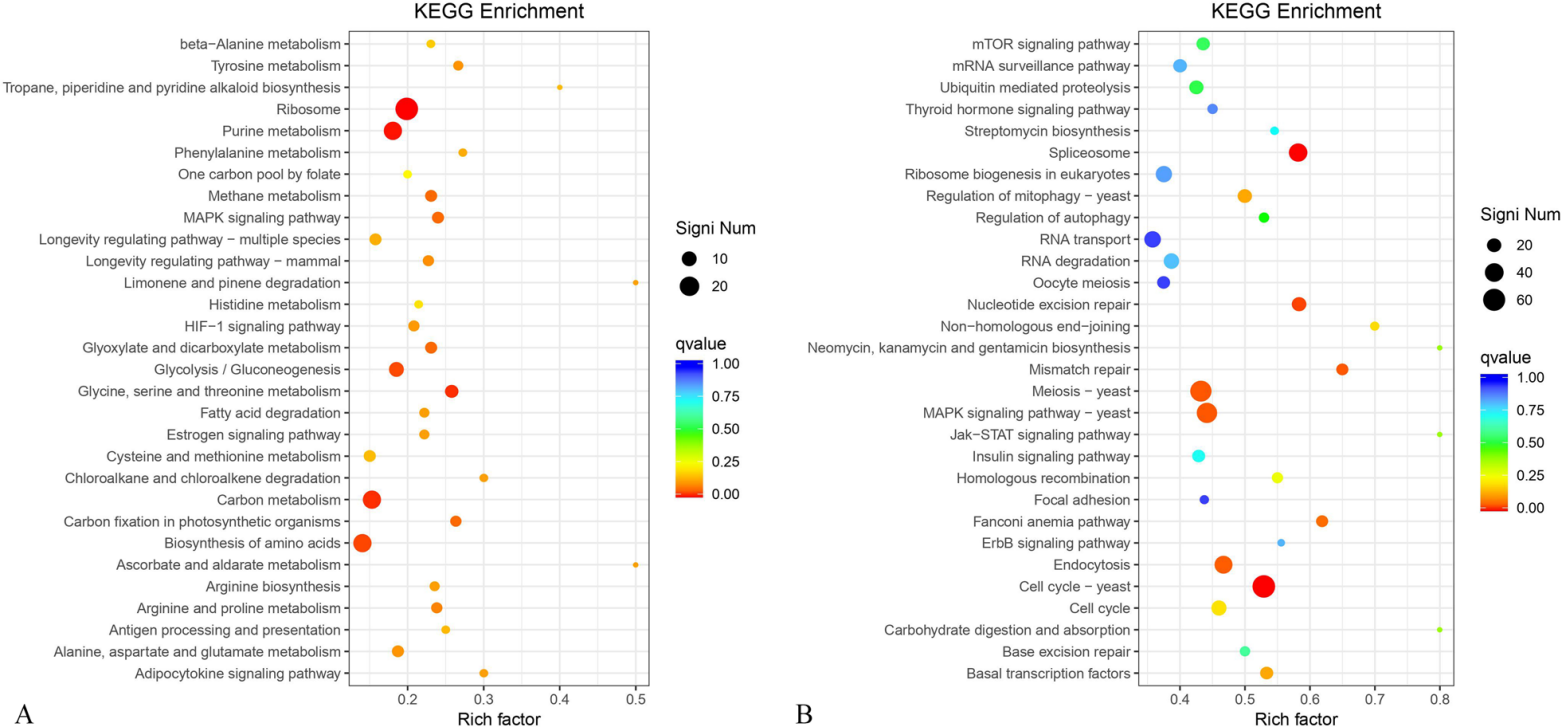
Up-regulated (**A**) and down-regulated (**B**) genes of *S. cerevisiae ERG5ΔERG4ΔERG3Δ* based on the KEGG enrichment pathway

Up-regulated and down-regulated genes were analyzed based on the significantly different functions using the KEGG enrichment pathway (Fig. 8). KEGG enrichment indicated the percentage of up-regulated genes mainly accounted for 20–30% of corresponding functions (Fig. 8A). The largest two proportions were from functional categories of “ascorbate and alkaline metabolism” (49%) and “Limonene and pinene degradation” (50%). The functional category “ribosome” had the most significantly different genes (29) among the tested functions. The percentage of down-regulated genes mainly accounted for 40–60% of the tested 30 functions (Fig. 8B). Function “yeast cell cycle” possessed the most differential genes (64) among the functions. Down-regulated genes involving the mTOR signaling pathway accounted for 43% of the corresponding function. mTOR signaling pathway was composed of mTOR complex 1 (mTORC1) and mTORC2. TORC1 was an important regulator of cell responses to different stresses such as high temperature.

### Steroid biosynthesis in *S. cerevisiae ERG5ΔERG4ΔERG3Δ*

Steroid biosynthesis from terpenoid backbone biosynthesis in *S. cerevisiae ERG5ΔERG4ΔERG3Δ* is drawn in Additional file 2. After multi-step catalytic reactions from initial terpenoid backbone biosynthesis, the main products flowed to vitamin D2, phytosterol, and primary bile acid biosynthesis for further metabolism. During the metabolism of ergosterol, up-regulated genes of *ERG1*, *ERG11*, and *ERG5* and down-regulated genes of *ERG9* and *ERG26* in *S. cerevisiae ERG5ΔERG4ΔERG3Δ* were tested based on the KEGG pathway analysis. The results showed that *ERG5*, *ERG4*, and *ERG3* gene deletion resulted in significant expression differences of genes involving the steroid metabolic pathway based on transcriptomics.

### Corn ethanol production by *S. cerevisiae ERG5ΔERG4ΔERG3Δ* at 37 °C

**Fig. 9:**
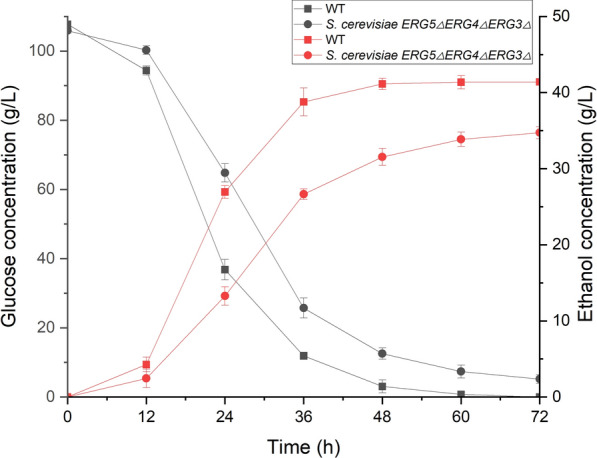
Ethanol and corn liquefied glucose concentrations during the fermentation of *S. cerevisiae ERG5ΔERG4ΔERG3Δ* at 37 °C

*S. cerevisiae ERG5ΔERG4ΔERG3Δ* was used to produce ethanol at 37 °C with corn as material using the industrialized production processes. The concentrations of ethanol and sugar were measured during the fermentation of corn saccharified liquid (Fig. 9). *S. cerevisiae ERG5ΔERG4ΔERG3Δ* produced ethanol concentrations of 41.6 g/L in a 20-L fermentor after fermentation for 60 h at 37 °C using the initial corn liquefied glucose concentration of 107.74 g/L. The conversion rate of sugar and ethanol from *S. cerevisiae ERG5ΔERG4ΔERG3Δ* achieved 0.386 g ethanol/g corn liquefied glucose. The ethanol concentration of the wild-type strain was 33.86 g/L with the glucose and ethanol conversion rate of 0.344 g/g after fermentation for 60 h at 37 °C. The ethanol yield of engineered strain increased by 12.21% in comparison with the wild-type strain. The results indicated that thermotolerant *S. cerevisiae ERG5ΔERG4ΔERG3Δ* had potential in large-scale industrial ethanol production from grain sugar under high-temperature conditions.

## Discussion

The development of thermotolerant *S. cerevisiae* has attracted considerable attention in the simultaneous saccharification and fermentation of lignocellulose materials via enzymatic hydrolysis coupled with ethanol production in the second-generation bioethanol [4]. In a first-generation ethanol industry, the improvement of temperature tolerance of *S. cerevisiae* was still of great significance in saving energy and reducing pollution in the fermentation process [25]. The fermentation efficiency of *S. cerevisiae* is very low under conditions of more than 35 degrees Celsius due to an increase in fluidity in membranes [26]. The thermotolerance acquisition of yeast was mainly controlled by specific stress-related genes involved in the specific compound in cell membranes that protected *S. cerevisiae* from high temperatures [27]. Ergosterol and other intermediate metabolites were essential components in membranes that determined the fluidity and permeability of membrane-associated proteins and affected resistance to external stress. Ergosterol was formed via a complex pathway involving the participation of many enzymes including Erg3, Erg4, and Erg5 [20]. The alteration of sterol composition improved the thermotolerance of yeast [21]. As a less toxic ergosterol intermediate, 14-methyl-fecosterol accumulated in *S. cerevisiae erg3* mutant [28]. Ergosta-5,7,24(28)-trienol formed from fecosterol and episterol in presence of Erg3 was converted into Ergosta-5,7,22,24(28)-trienol, which was a catalytic product of Erg5 and further converted into ergosterol by Erg4 [20]. In this study, simultaneous deletion of engineered *S. cerevisiae ERG3*, *ERG4*, and *ERG5* resulted in improved thermotolerance. In addition, engineered *S. cerevisiae ERG3*, *ERG4*, and *ERG5* achieved high-temperature fermentation at 37 °C to produce corn ethanol according to the large-scale industrialized production processes (Fig. 1). The deletion of *ERG3*, *ERG4*, and *ERG5* in *S. cerevisiae* caused the expression disturbance of multiple genes involving in de novo ergosterol synthesis based on the KEGG analysis. In addition, this study further proved the practical application value based on the theory of sterol composition alteration boosting yeast thermotolerance proposed by Caspeta et al. [21].

Not only gene deletion in yeast resulted in the thermotolerance improvement of yeast, but also gene overexpression in engineered yeasts could cause advantageous application in *Eucalyptus globulus* wood and corn cob hydrolysates [29]. For instance, *PRS3*, *RPB4*, and *ZWF1* overexpression in two industrial *Saccharomyces cerevisiae* strains increased adaptation performance [29]. In this study, a general laboratory strain S288C was used as a host to increase its thermotolerance with an optimal culture temperature of 30 °C. Industrial *S. cerevisiae* Ethanol Red has an optimum temperature of 35 °C. The overexpression of Erg13 encoding a protein involved in early ergosterol biosynthesis and Gsy1 encoding a glycogen synthase contributed to yeast adaptation to thermotolerance. In addition, the cumulative content of trehalose after Erg13 and Gsy1 overexpression was also higher than that of other strains [5, 6]. These studies provided references for construction of thermotolerant engineered strain by overexpression of specific genes.

## Conclusions

Various deletion combinations of *ERG5*, *ERG4*, and *ERG3* using CRISPR–Cas9 were investigated to boost the thermotolerance of engineered *S. cerevisiae* for ethanol production. Engineered *S. cerevisiae ERG5ΔERG4ΔERG3Δ* possessed the highest thermotolerant capability among engineered *S. cerevisiae* strains. Engineered *S. cerevisiae ERG5ΔERG4ΔERG3Δ* represented 278 up-regulated genes and 1892 down-regulated genes in comparison with the wild-type strain. *ERG5*, *ERG4*, and *ERG3* gene deletion resulted in significant expression differences of genes involving steroid metabolic pathway based on transcriptomics. The conversion rate of corn liquefied glucose and ethanol of *S. cerevisiae ERG5ΔERG4ΔERG3* was 0.386 g ethanol/g glucose at 37 °C. Engineered *S. cerevisiae ERG5ΔERG4ΔERG3Δ* has potential in large-scale industrialized applications for ethanol production using starchy grain under high-temperature conditions.

## Materials and methods

### Plasmids, strains, primers, and agents

*S. cerevisiae* S288c (WT) used in this experiment was a haploid strain. The plasmid gRNA-trp-HyB carrying hygromycin B resistance gene for guide RNA synthesis and Cas9-NTC carrying nourseothricin resistance gene for *S. cerevisiae* genomic DNA digestion were from Addgene (Watertown, Massachusetts, USA). *Escherichia coli* DH5α for plasmid amplification and enzymes were from Sangon Biotech (China). The primer synthesis and gene sequencing were performed by Sangon Biotech (China). All biochemical reagents are analytically pure.

### gRNA vector synthesis

To construct the gRNA vector of *S. cerevisiae ERG3*, *ERG4*, and *ERG5* target genes, 20-bp nucleotide sequences were obtained based on the efficiencies of target sequences from website (http://chopchop.cbu.uib.no/system) for CRISPR/Cas9 knocking-out. Three pairs of primers containing target sequences and gRNA-trp-HyB sequences were synthesized by Sangon Biotech (China). The prepared primers were used to amplify the plasmid gRNA-trp-HyB to obtain the corresponding linear vectors for the gRNA target (Table 1). High-fidelity PCR master mix containing Phusion DNA polymerase was used to amplify target genes in a 25-μL reaction system with parameters of 95 °C preheating for 2 min, 95 °C for 15 s, 56 °C for 15 s, 72 °C for 3 min, 35 cycles. The amplified gRNA vectors were extracted, purified, and stored for transformation.

**Table 1:** Prepared primers for amplification of genes and plasmids

Primers	Sequences (5′ → 3′)	Sizes
ERG5-gRNA-F1	ATTTCATGGAAAAAGACCTGGGGGTTTTAGAGCTAGAAATAGCAAG	6509
ERG5-gRNA-R1	CCCCAGGTCTTTTTCCATGAAATGATCATTTATCTT TCACTGCGGA	bp
ERG4-gRNA-F1	CTATGTGACACCACAATTGGGGGGTTTTAGAGCTAGAAATAGCAAG	6509
ERG4-gRNA-R1	CCCCCAATTGTGGTGTCACATAGGATCATTTATCTTTCACTGCGGA	bp
ERG3-gRNA-F1	AAGATTGATTATGAAAACCACGGGTTTTAGAGCTAGAAATAGCAAG	6509
ERG3-gRNA-R1	CCGTGGTTTTCATAATCAATCTTGATCATTTATCTTTCACTGCGGA	bp
dDNA-MFC-F	GTTCCGTATCGCACACGCCG	628
dDNA-MFC-R	CTAGCTAACATTAATGTTGA	bp
dDNA-XYNA-F	CCCCACACACCATAGCTTCA	1129
dDNA-XYNA-R	GCGGATGTGGGGGGAGGGC	Bp
dDNA-CEL-F	CCCCACACACCATAGCTTCA	880
dDNA-CEL-R	CCGCCTGCGCCGCTCCGGTG	bp

ERG5-gRNA, ERG4-gRNA, and ERG3-gRNA primers were used to construct the vectors for ERG5-gRNA, ERG4-gRNA, and ERG3-gRNA, respectively. Three pairs of primers for the amplification of *MFC* (*Ampullaria crossean multi-functional cellulase*, 628 bp), *XYNA* (*Endo-1,4-beta-xylanase*, 1129 bp), and *CEL* (*Cellulase*, 880 bp) were used to knock-out *ERG5*, *ERG4*, and *ERG3* as donor DNA, respectively

### *S. cerevisiae ERG4*, *ERG5*, and *ERG3* deletion using the CRISPR–Cas9 approach

*S. cerevisiae* transformation in this study was carried out using the reported LiAc/SS DNA/PEG approach [30]. Each *S. cerevisiae* gene knock-out was performed using two-step transformation: Cas9-NTC transformation on the screening media containing 80 μg/mL of nourseothricin, and then further integration of linear gRNA vector on the double-antibiotic screening medium containing 300 μg/mL of hygromycin B and 80 μg/mL of nourseothricin. The putative transformants were selected and confirmed after sequencing of inserted DNA. In this study, *S. cerevisiae ERG3ΔERG4ΔERG5Δ* mutation strain was obtained after *S. cerevisiae ERG4*, *ERG5*, and *ERG3* genes were knocked out in turn (Fig. 2A).

### Growth and thermotolerance test of engineered *S. cerevisiae*

The growth profile of engineered *S. cerevisiae *was determined according to the optical density (OD) at the wavelength of 600 nm. The thermotolerance of *S. cerevisiae* was tested on the solid and liquid YPD media. The wild-type and engineered *S. cerevisiae* solution were diluted by gradient and transferred to the solid YPD plate for culture at 37 °C and 42 °C. The viability assessment of *S. cerevisiae* was performed under an optical microscope using the methylene-blue dyeing method [31]. The life or death of strain was reflected based on the staining degree of the cell wall. The mortality percentages of wild-type and engineered *S. cerevisiae ERG5ΔERG4ΔERG3Δ* were compared to analyze the effect of gene deletion on the thermotolerance of *S. cerevisiae*.

### Glucose consumption and ethanol production of engineered *S. cerevisiae*

The effect of different temperatures of 30, 37, and 42 °C on the glucose consumption and ethanol production of engineered *S. cerevisiae ERG5ΔERG4ΔERG3Δ* were investigated using the wild-type strain as the control. The high-performance liquid chromatography (HPLC) method was used to measure the glucose and ethanol contents as the following parameters of Waters 2410 Refractive Index detector, Shodex SH1011 chromatographic column, a mobile phase of 0.01 mol/L H_2_SO_4_, a flow rate of 0.6 mL/min, injection volume of 5 μL, and column temperature of 50 °C.

### Transcriptomic analysis

The cell proliferations of engineering *S. cerevisiae* and the wild-type strains were carried out under the liquid culture conditions at 30 ℃. *S. cerevisiae* cDNA library was constructed via the processes of total RNA extraction, mRNA purification, and cDNA synthesis. cDNA was subjected to end A tail addition. Linker ligation reactions were finally performed [32]; the products from cDNA library were purified using Hieff NGS™ DNA Selection Beads; then, the constructed cDNA library was sequenced using the Illumina Hiseq™ approach [33]. Transcriptomics on the mRNA level was further analyzed based on the sequenced data above. The original data file from Illumina Hiseq™ was recorded by CASAVA. After N bases, linker sequences in reads, and low-quality sequences (*q*-value < 20) were removed by a Trimmomatic tool, and the clean data were obtained for use [34, 35]. After quality control, the obtained sequences were compared with those from the S288c genome as a reference by HISAT2 [36]. After the count of results using the RSeQC method [37], Transcripts Per Million (TPM) was applied to estimate the samples. Differential expression gene (DEG), Gene Ontology (GO), and Kyoto Encyclopedia of Genes and Genomes (KEGG) were used to analyze gene differences, annotate, and define gene functions [38, 39].

### Corn ethanol production of *S. cerevisiae ERG5ΔERG4ΔERG3Δ* at 37 °C

Corn was used to produce ethanol by engineered *S. cerevisiae ERG5ΔERG4ΔERG3Δ* strain in a 20-L fermentation tank using fed-batch fermentation technology. The ethanol production of engineered *S. cerevisiae ERG5ΔERG4ΔERG3Δ* was used to evaluate the application feasibility under a high-temperature condition of 37 °C. The detailed processes were as follows: (1) raw material crushing. Dry corn as raw material after being crushed into fine powder was mixed with amylase and water; (2) liquefaction treatment. The uniform solution was heated and transported into a liquefaction tank to enter the liquefaction process; (3) fermentation. The liquefied solution was transferred into the fermentation tank after cooling. Then, the fermented mature solution containing ethanol was obtained after fermentation; (4) distillation. The fermented mature solution was sent into a distillation tower for the purification and dehydration of ethanol (Additional file 3).

### Data analysis and figure processing

All statistics data from three repetitions were represented using mean ± standard error in this study. OrginPro 2018 and Adobe Photoshop CC 2018 were used to draw curve figures and design image typesetting, respectively.

## Supplementary Information

**Additional file 1. **Gene expression density distribution (A) and DEG analysis (B) between *S. cerevisiae ERG5ΔERG4ΔERG3Δ* and wild-type strain.

**Additional file 2. **Steroid biosynthesis modification of *S. cerevisiae ERG5ΔERG4ΔERG3Δ* from terpenoid backbone biosynthesis after gene deletion.

**Additional file 3. **Technical route of ethanol production from corn by liquefaction, high-temperature fermentation, and distillation.

**Additional file 4. S. cerevisiae** DEGs.

## Data Availability

All the data generated in the study are included in the present manuscript. In addition, the data of DEGs are listed in Additional file 4.
