# Supplementary figures and images for: Thermotolerance improvement of engineered Saccharomyces cerevisiae ERG5 Delta ERG4 Delta ERG3 Delta, molecular mechanism, and its application in corn ethanol production
Source: Biotechnol Biofuels Bioprod. 2023 Apr 12;16:66. doi: 10.1186/s13068-023-02312-4 (PMC10091661; doi:10.1186/s13068-023-02312-4)

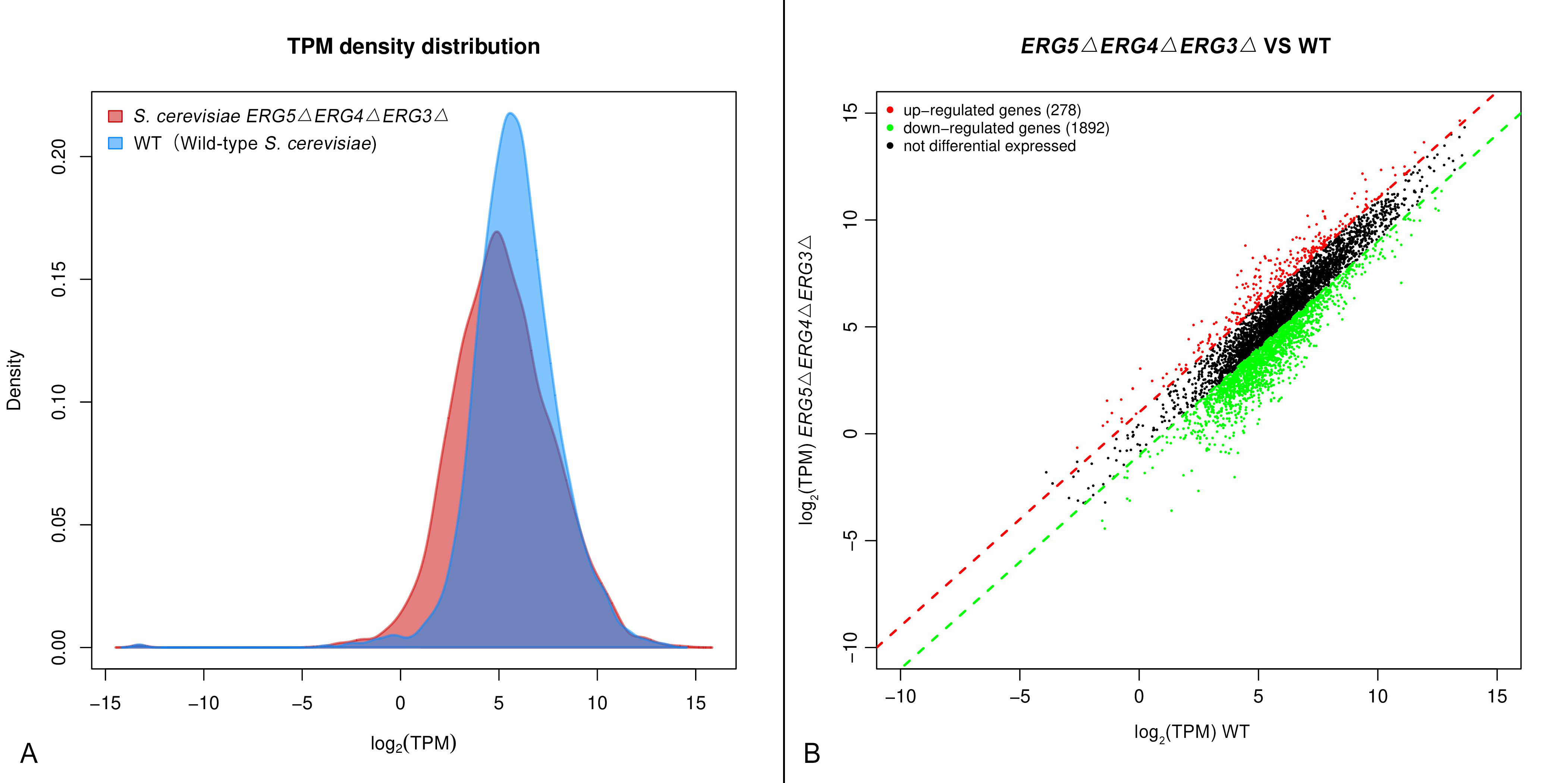

Supplement: Supplementary file 1 — Additional file 1. Gene expression density distribution (A) and DEG analysis (B) between S. cerevisiae ERG5ΔERG4ΔERG3Δ and wild-type strain. [file 13068_2023_2312_MOESM1_ESM.jpg]

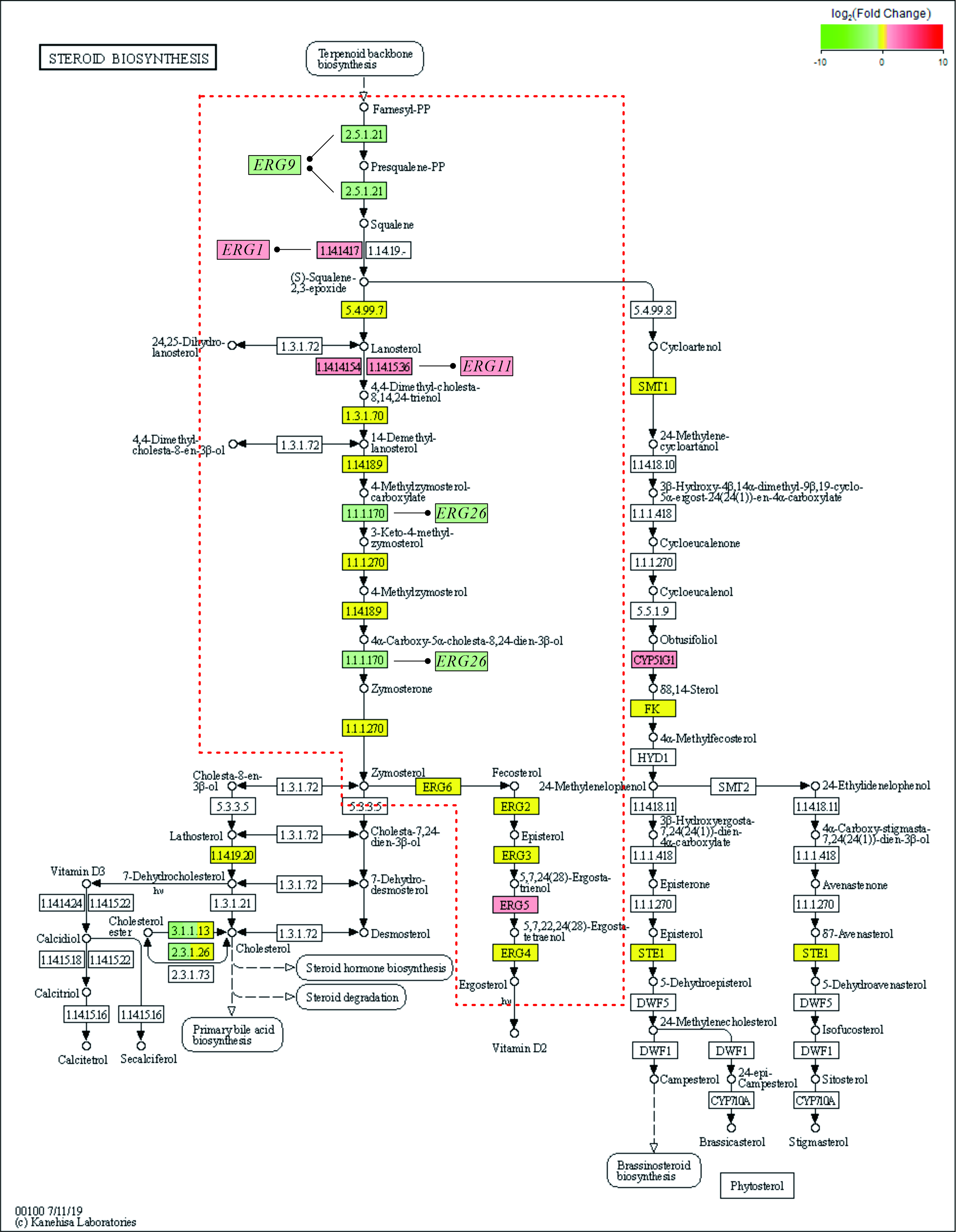

Supplement: Supplementary file 2 — Additional file 2. Steroid biosynthesis modification of S. cerevisiae ERG5ΔERG4ΔERG3Δ from terpenoid backbone biosynthesis after gene deletion. [file 13068_2023_2312_MOESM2_ESM.jpg]

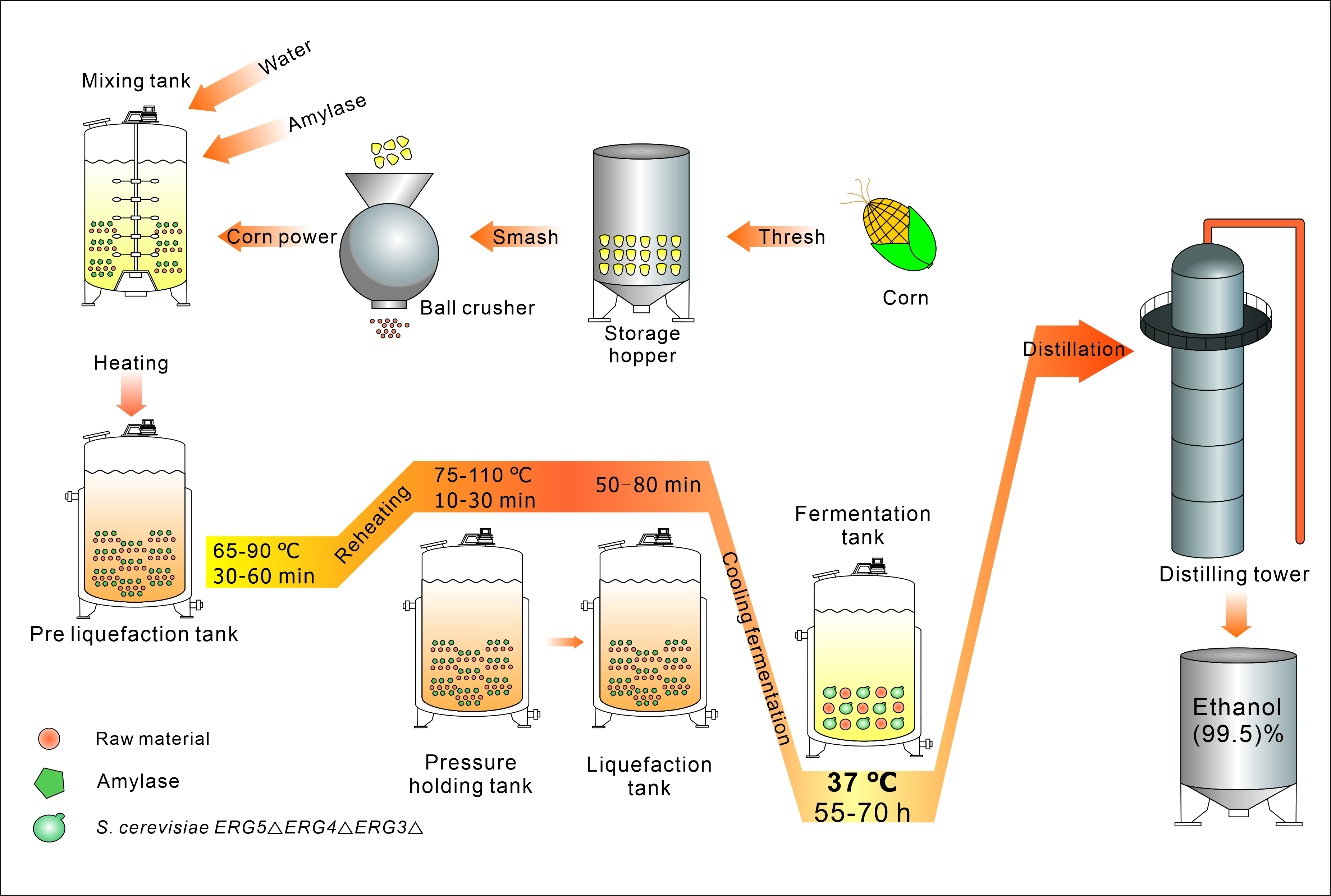

Supplement: Supplementary file 3 — Additional file 3. Technical route of ethanol production from corn by liquefaction, high-temperature fermentation, and distillation. [file 13068_2023_2312_MOESM3_ESM.jpg]
